# Bi-Factor Analysis Based on Noise-Reduction (BIFANR): A New Algorithm for Detecting Coevolving Amino Acid Sites in Proteins

**DOI:** 10.1371/journal.pone.0079764

**Published:** 2013-11-20

**Authors:** Juntao Liu, Xiaoyun Duan, Jianyang Sun, Yanbin Yin, Guojun Li, Lushan Wang, Bingqiang Liu

**Affiliations:** 1 School of Mathematics, Shandong University, Jinan, China; 2 School of Life Science, Shandong University, Jinan, China; 3 Department of Biological Sciences, Northern Illinois University, DeKalb, Illinois, United States of America; Rosalind Franklin University, United States of America

## Abstract

Previous statistical analyses have shown that amino acid sites in a protein evolve in a correlated way instead of independently. Even though located distantly in the linear sequence, the coevolved amino acids could be spatially adjacent in the tertiary structure, and constitute specific protein sectors. Moreover, these protein sectors are independent of one another in structure, function, and even evolution. Thus, systematic studies on protein sectors inside a protein will contribute to the clarification of protein function. In this paper, we propose a new algorithm BIFANR (Bi-factor Analysis Based on Noise-reduction) for detecting protein sectors in amino acid sequences. After applying BIFANR on S1A family and PDZ family, we carried out internal correlation test, statistical independence test, evolutionary rate analysis, evolutionary independence analysis, and function analysis to assess the prediction. The results showed that the amino acids in certain predicted protein sector are closely correlated in structure, function, and evolution, while protein sectors are nearly statistically independent. The results also indicated that the protein sectors have distinct evolutionary directions. In addition, compared with other algorithms, BIFANR has higher accuracy and robustness under the influence of noise sites.

## Introduction

The amino acids coevolution is very common in various protein families [1], [2], [3]. Highly conserved amino acid sites are often located in the core or on the functional surface of protein tertiary structure [4], [5], [6]. These sites usually are under strong evolutionary constraint, thus are critical for maintaining the protein’s function. The amino acid sites that are highly correlated in evolution often form protein sectors [7], [8]. Protein sectors decompose proteins into quasi-independent groups, which are distinct from the traditional hierarchy of protein structure. The statistical characteristic analysis of the cooperative action of conserved amino acids could contribute to the inference of protein function and evolution [1], [9].

Since functionally important amino acid regions in a protein are usually conserved in evolution, researchers have been identifying these regions by performing directed mutagenesis experiments [10], [11], [12], [13]. However, such experimental approaches are time and labor intensive. In order to overcome this problem, researchers have developed statistical methods to detect functionally dependent (or correlated) amino acids in proteins using coevolution analysis [14], [15]. For example, some parametric and non-parametric methods were employed to detect important amino acid sites [16], [17], [18], [19], [20], [21], [22], which usually focus on amino acids important for maintaining the protein structure and function. These methods rely on multiple sequence alignment (MSA), so the quality and size of MSA and the background coevolution noise became the main obstacles [15], [18], [23]. In addition, some other typical probabilistic models have also been implemented, e. g. Maximum likelihood approximation [24], [25], [26], Bayesian probabilities [27], phylogenetic approaches [28] and sequence divergence based approximation [15], [29]. Lastly, several new ideas were introduced to reduce the influence of noise [7], [8]. However, these methods can only reach high accuracy in some specific protein families, thus cannot be widely used. Therefore, there is a need of more effective method to be developed.

**Figure 1:**
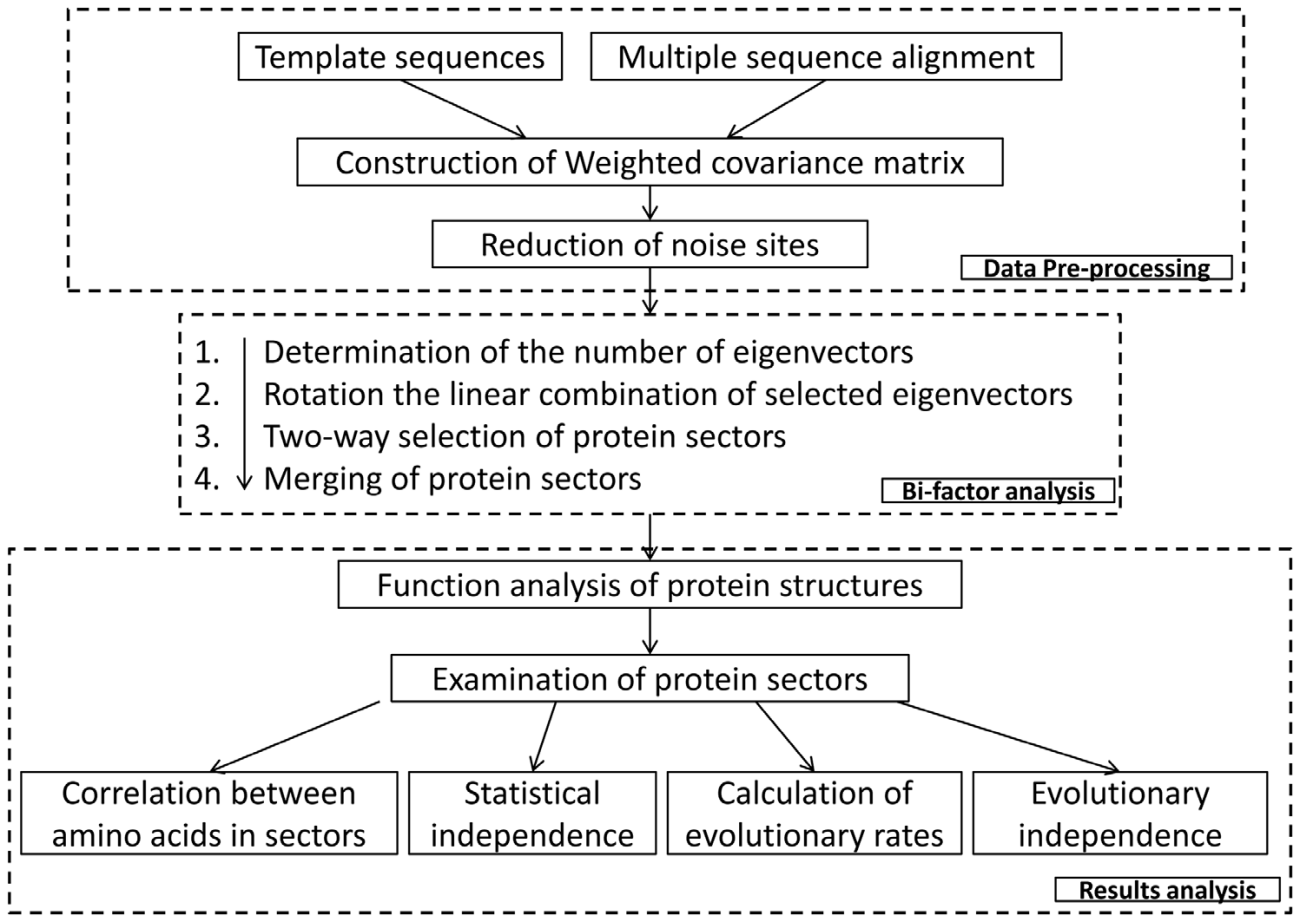
Methods flowchart.

In this paper, we propose a new algorithm, named BIFANR (Bi-factor analysis based on noise-reduction), to reveal the coevolving pattern of amino acid sites. The algorithm originates from the Factor Analysis in psychological researches [30], which is widely used to analyze psychological factors, such as human personality and sensibility. Like previous studies, our algorithm follows the following principals: 1) the coevolved amino acid sites in a protein constitute a protein sector, which are closely combined in the tertiary structure to account for certain biological characteristics; 2) different protein sectors are independent of each other in terms of the tertiary structure and function. However, different from other methods, BIFANR first conducts noise reduction before factor analysis, which improves efficiency and accuracy. After that, a bi-factor analysis is employed to determine the corresponding eigenvectors of non-random eigenvalues with a stochastic simulation and then to extract protein sectors. In linear combination of eigenvectors, this algorithm employs varimax orthogonal rotation to ensure independence between protein sectors. Furthermore, we applied BIFANR to a PDB structure 3TGI of the S1A serine protease family and 1BE9 of the PSD95/Dig1/ZO1 (PDZ) family. As a result, we found 3 protein sectors in 3TGI and 2 in 1BE9. Further analysis showed that BIFANR has higher accuracy and robustness compared with other algorithms. The flowchart of the complete analyses is presented in Figure 1. The source code of the BIFANR program is available in the file Program S1.

## Results

### 1. The Algorithm Design of BIFANR

The BIFANR algorithm consists of two major components, detailed as follows:

#### 1.1 Correlation coefficient matrix and noise reduction

This algorithm applies the idea of Factor Analysis to amino acid site analysis to extract protein sectors. Specifically, starting from a given MSA, we first calculated the correlation coefficients between amino acid sites and constructed a covariance matrix (non-weighted correlation coefficient matrix, see **Methods**). Considering the biological significance, we then gave weights to the covariance matrix like previous studies [7], [31]. Finally, we calculated the weighted correlation coefficient matrix based on the background frequency of the 20 amino acids and the conservation of amino acid sites. As a result, we have measured the pair-wise correlation of amino acid sites with this matrix, based on which we further conducted noise reduction.

The noise sites are amino acid sites that are weakly correlated with almost all the other sites. These noise sites usually reduce the efficiency and accuracy of the algorithm to identify protein sectors. This is the main reason causing the failure of some covariance amino acid sites detecting methods [32]. In order to overcome this problem, we developed a noise reduction method in the pre-processing step. Specifically, the amino acid sites with high randomness in evolution are removed before the detection step.

Taking S1A family and PDZ family as examples: in the S1A family, there were 223 sites in the multiple sequence alignment (MSA) of 3TGI [33] and its homologous protein sequences and 104 sites were removed by the noise reduction step; in the PDZ family, 49 sites of the total 94 sites were removed after the noise reduction step. These removed sites are weakly correlated with other sites, and have higher evolutionary rates than the remaining sites. Calculated by Rate4Site, in S1A family [34], the average evolutionary rates of removed and remained sites are 0.7692 and −0.6723 respectively. In PDZ family they are 0.6717 and −0.7314 respectively.

#### 1.2 Bi-factor analysis

In the bi-factor analysis, we obtained protein sectors according to the eigenvectors of the weighted correlation coefficient matrix. In order to guarantee the non-randomness of the predicted protein sectors, we simulated the data by randomly shuffling the multiple sequence alignment for 100 times, and then chose the non-random eigenvectors of the correlation coefficient matrix based on the stochastic simulation result. Using this method, we can find the non-random protein sectors hidden in the protein sequence. The original factor coefficient for each amino acid site can be considered as the correlation between the site and the factor (i.e. selected eigenvectors of the correlation coefficient matrix, see Methods). BIFANR assigns amino acid sites to factors according to the correlation. However, we cannot obtain protein sectors based on the original factor coefficients directly because one site may have similar coefficients with different factors. Thus we have further conducted varimax orthogonal rotation for these factors. Our ideal expectation is that each site will have a large coefficient value with just one factor, which could be sufficient to distinguish this factor from the remaining factors. Consequently, the protein sectors detected by BIFANR will have significant statistical independence.

After varimax orthogonal rotation, amino acid sites were assigned to factors according to the factor coefficients calculated above. As the coefficient is within the range [−1, 1], there are both positive and negative correlation and the larger the absolute value is, the more significant the correlation is. Those sites might also form a protein sector, if they have significant negative correlation with one factor. Therefore, BIFANR conducted bidirectional selection of factor coefficients on the basis of factor analysis, which could prevent the loss of protein sectors due to solely selection of positive factor coefficients. However, bidirectional selection may cause the occurrence of two overlapping protein sectors. In order to merge overlapping protein sectors, we retain the overlap and then add those sites having higher correlation with current sites.

### 2. Statistical and Biological Tests for Protein Sectors

BIFANR detected three protein sectors in S1A family and two protein sectors in PDZ family. To verify these protein sectors and evaluate the performance of our algorithm, we conducted statistical tests and did biological analysis with these protein sectors. The statistical tests include internal correlation test and statistical independence test. Besides, we conducted evolutionary rate test.

**Figure 2:**
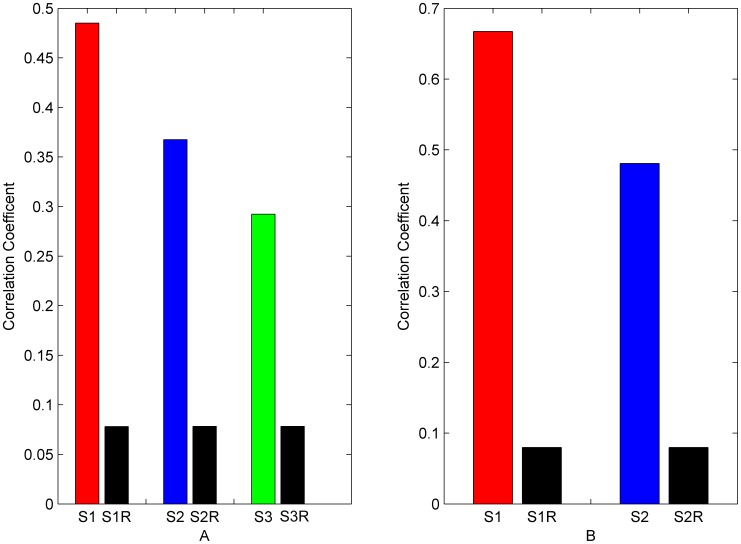
Average correlation coefficients of protein sectors. A: Average correlation coefficient of each protein sector in S1A family. B: Average correlation coefficient of each protein sector in PDZ family. Red column represents average correlation coefficient of protein sector 1. Blue column represents average correlation coefficient of protein sector 2. Green column represents average correlation coefficient of protein sector 3. Black column represents stochastic expected average correlation coefficient of each protein sector.

To demonstrate the correlation between the amino acid sites within a protein sector, we took all of the amino acid sites in each protein sector and calculated the mean correlation coefficients between each pair of the amino acid sites. In addition, we randomly simulated the data set with the same number of sites for 1000 times and similarly calculated the mean correlation coefficients to be the random expectation. The results showed that in each protein sector, the average of correlation coefficients is much higher than the random expectation (Figure 2).

**Figure 3:**
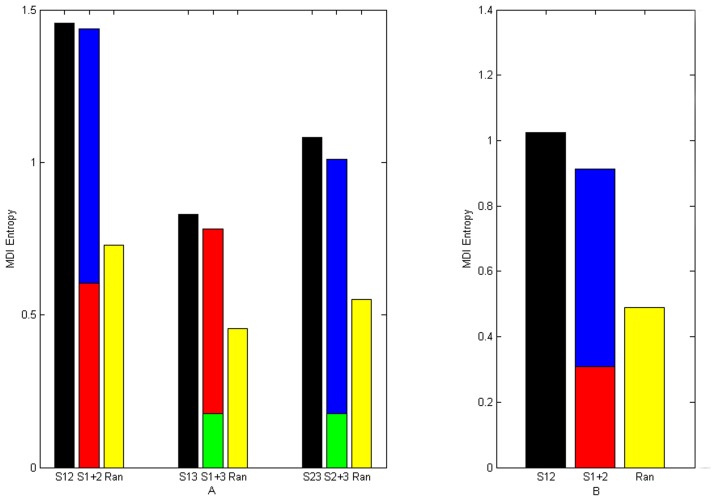
Statistical independence of protein sectors. A: Statistical independence of protein sectors in S1A family. B: Statistical independence of protein sectors in PDZ family. (Red column represents MDI entropy of protein sector 1. Blue column represents MDI entropy of protein sector 2. Green column represents MDI entropy of protein sector 3. Black column represents MDI entropy of two protein sectors as a whole. Yellow column represents stochastic expected MDI entropy after disrupting the amino acid sites within two protein sectors 100 times.).

To illustrate the statistical independence between protein sectors, we calculated the MDI entropy of S1A family and PDZ family, respectively. The MDI entropy was originally used to quantify the degree to which a selected group of amino acid sites are statistically coupled to each other in an MSA. If two protein sectors are independent, the MDI entropy of them taken together should be equal to the sum of their MDI entropies taken individually in theory. The results supported this conjecture by showing that the MDI entropy of each two predicted protein sectors was much higher than the random expectation, which means that the protein sectors are statistically independent of each other (Figure 3).

**Figure 4:**
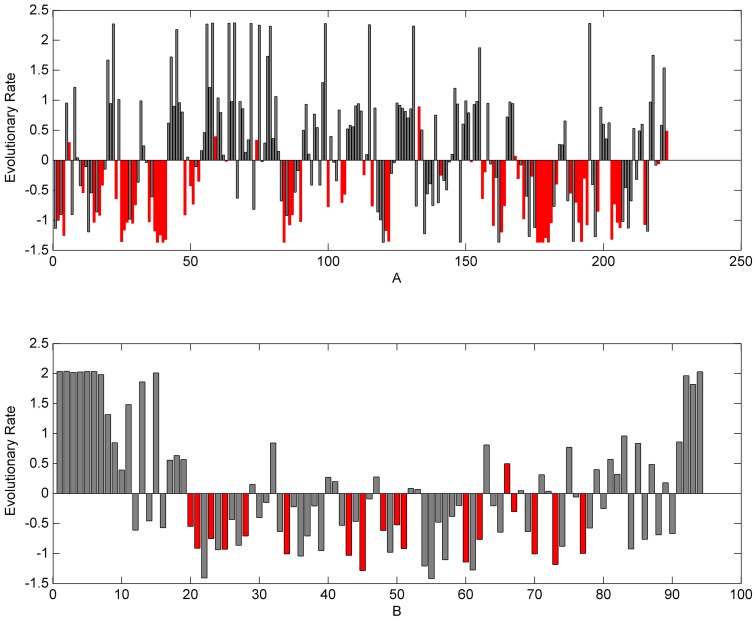
Distribution of amino acid site evolutionary rates. A: Distribution of amino acid site evolutionary rates in S1A family (rat trypsin: 3TGI). B: Distribution of amino acid site evolutionary rates in PDZ family (rat PSD-95∶1BE9). Red column represents the evolutionary rate distribution of amino acid sites in protein sectors.

To study the evolutionary feature of amino acid sites within a protein sector, we calculated the average evolutionary rates of the amino acid sites in the entire protein and the amino acid sites in the protein sectors, respectively. The results showed that the latter was much lower than the former (Table 1). Figure 4 shows the result for both the S1A family and the PDZ family, where for both families, the evolutionary rates of over 90% sites in protein sectors are negative, suggesting that these sites have lower evolutionary rates and thus are selectively constrained to maintain the protein structure and function.

**Table 1:** Average evolutionary rate of each protein sector and average evolutionary rate of all sites in S1A and PDZ.

	sector 1	sector 2	sector 3	all sites
**S1A**	−0.7288	−0.4520	−1.2143	0
**PDZ**	−0.8683	−0.9163	–	0

### 3. Comparison to Buck’s Method

In order to evaluate the performance of BIFANR, we compared our method with Buck’s method [8]. In comparison, we chose 3TGI of the S1A family and 1BE9 [35] of the PDZ family as template sequences, since protein sectors in these two template sequences have been experimentally verified [7]. Then we evaluated the predicted results of the two methods by comparing experimentally confirmed sectors with our predicted sectors (i.e. factors in [8] ). If all or most sites of an experimentally confirmed sector are found in just one predicted sector, it means that the prediction is reliable. Otherwise, if sites are found in several predicted sectors, it indicates that the prediction is unreliable. We then calculated the percentage of experimentally confirmed sectors that are found in our predicted sectors, i.e. sensitivity and the percentage of our predicted sectors to be true positives (i.e. experimentally confirmed sectors), i.e. positive predictive value (PPV).

**Figure 5:**
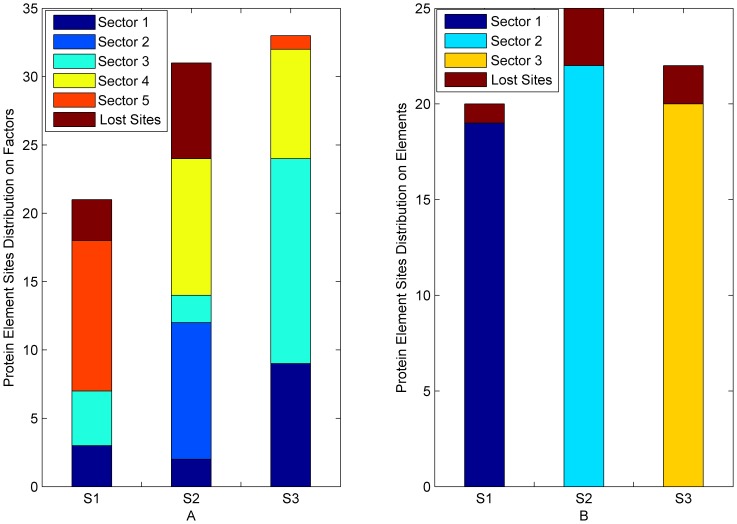
Comparison between BIFANR and Buck’s in S1A family. A: S1,S2, and S3 represent 3 experimental confirmed protein sectors in S1A family and the height of color bar represents the number of sites in corresponding predicted protein sector by Buck’s method. B: S1,S2, and S3 represent 3 protein sectors in S1A family and the height of each color bar represents the number of sites in corresponding predicted sector by BIFANR. And the height of the brown bar represents the number of lost sites by algorithms in each experimental confirmed protein sector.

**Figure 6:**
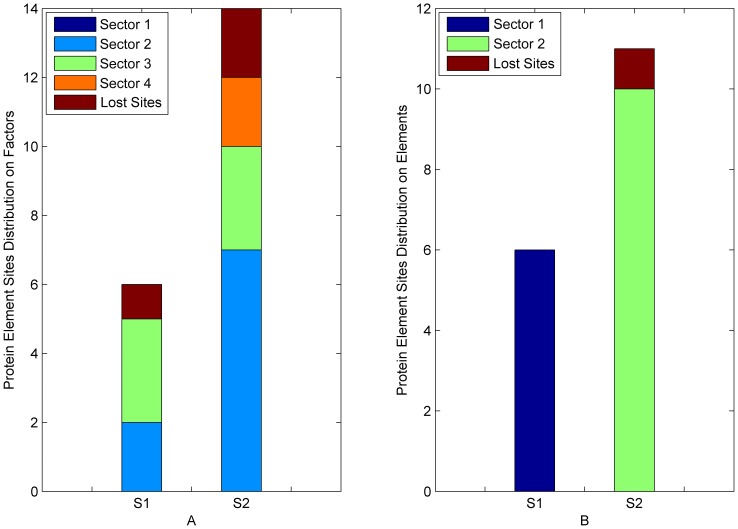
Comparison between BIFANR and Buck’s in PDZ family. A: S1 and S2 represent 2 experimental confirmed protein sectors in PDZ family and the height of each color bar represents the number of sites in corresponding predicted protein sector by Buck’s method. B: S1 and S2 represent 2 protein sectors in PDZ family and the height of each color bar represents the number of sites in corresponding predicted sector by BIFANR. And the height of the brown bar represents the number of lost sites by algorithms in each experimental confirmed protein sector.

For the result of Buck’s method, the sites in experimentally confirmed sectors distributed almost uniformly in different predicted sectors. But for our result, the sites in any experimentally confirmed sector distributed on just one predicted sector (Figure 5, 6). The results show that the sensitivities of Buck’s method in S1A family and PDZ family were 85.07% and 82.35%, respectively, while those of our method were 91.04% and 94.11%. In addition, the PPVs of Buck’s method in S1A family and PDZ family were 43.84% and 29.16%, respectively, while those of our method were 90.77% and 94.11%. The results clearly demonstrate that BIFANR performs much better than Buck’s method.

### 4. Function Analysis of Protein Structure

**Figure 7:**
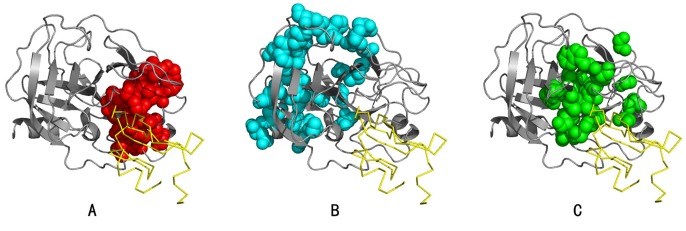
Protein sectors of S1A family (Rat trypsin PDB: 3TGI). A: Red balls represent protein sector 1, which mainly comprises residues located near the catalytic cleave. B: Blue balls represent protein sector 2, which comprises residues within the core of the two β barrels. C: Green balls represent protein sector 3, comprising residues within the catalytic cleave. Residues comprising protein sectors are displayed in space filling representation with a van der Waals surface.

BIFANR detected three and two protein sectors in S1A family and PDZ family respectively (see Table S1 for the amino acids in each protein sector). Strikingly, the amino acid sites in the three protein sectors of S1A family are not linearly close to each other in the sequence, but apparently are correlated in the tertiary structure (Figure 7A–C). In addition, protein sectors tend to independent of each other in protein function.

In S1A family, protein sector 1 mainly contains amino acids surrounding the pocket of S1 enzyme. Amino acid mutations within this sector may affect the substrate specificity of some enzymes in the family as residues in this sector is involved in transferring chymotryptic specificity into trypsin [36], [37], [38], [39], [40]. Protein sector 2 mainly contains amino acids of the two β-sheets in the protein core. The double alanine mutation in this sector could affect the thermal stability of the enzyme, but hardly affect the catalytic ability. Moreover, the mutations in this sector are synergistic. The effect of sector 1 to substrate specificity is independent on that of sector 2 to structure stability [7], [41], [42]. Protein sector 3, which is mainly responsible for catalytic ability, contains the catalytic triad (57H, 102D and 195S) and neighboring amino acids that are related to catalytic ability or accounting for allosteric regulation [36], [43], [44], [45]. This sector also includes one disulfide bond pair (42C–58C) and the substitution of this bond would cooperatively interact with mutation of S195. In addition, triple mutation of C42A, C58A/V, and S195T will convert trypsin from a serine protease to a threonine protease. So, this sector represents the catalytic core of this protease family.

**Figure 8:**
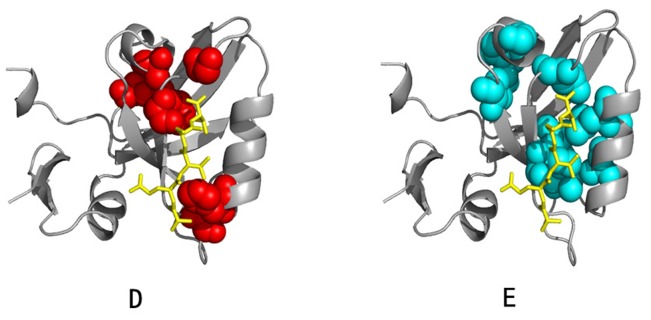
Protein sectors of PDZ family (Rat PSD-95 DPB: 1BE9). A: Red balls represent residues within protein sector 1. B: Blue balls represent residues within protein sector 2. Residues comprising protein sectors are displayed in space filling representation with a van der Waals surface.

In PSD95/Dig1/ZO1 (PDZ) protein domain family, protein sector 1 contains amino acids in α_2_–β_2_ groove and α_1_-helix, which affect the substrate binding affinity [31], [46] and the regulation of α_2_–β_2_ groove affinity [47]. The sites in this sector are either in relation to each other directly or are connected through interactions with the substrate peptides. In protein sector 2, residues 36 and 75 co-mutate to cysteine may be responsible for the redox-dependent equilibrium [48], [49] between two conformations in INAD PDZ5 [50] (Figure 8).

**Figure 9:**
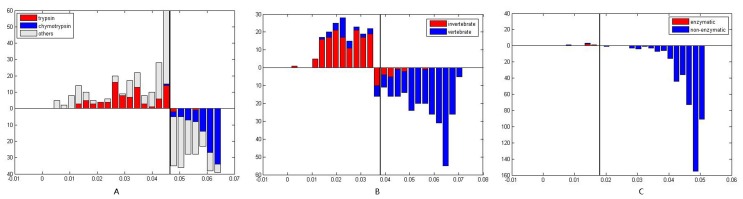
Evolutionary independence of protein sectors in S1A family. A: Evolutionary independence of protein sector 1. B: Evolutionary independence of protein sector 2. C: Evolutionary independence of protein sector 3.

To further investigate the function and independent evolution of protein sectors, we analyzed the evolutionary independence of the three protein sectors in S1A family. Evolutionary independence test is to construct a similarity matrix *M* with the sequence similarities of amino acid sites in a protein sector and then conduct principal component analysis. In principal component analysis, only one principal component was selected and all sequences were separated into two parts according to factor coefficients. Taking S1A family as an example, the results of the principal component analysis of protein sectors 1, 2 and 3 were displayed in Figure 9A, 9B and 9C, respectively. According to the sequence similarity of sites in each protein sector, casein (red, top) and chymotrypsin (blue, below) proteins are separated by protein sector 1 (Figure 9A); vertebrates and non-vertebrates are separated by protein sector 2 (Figure 9B); and enzymes and non-enzymes are separated by protein sector 3 (Figure 9C). These results indicate that protein sector 1 may be responsible for the specificity of substrate recognition in the catalytic process; protein sector 2 may be involved in the protein backbone evolution, while protein sector 3 may account for the protein catalytic activity.

### 5. Application to Hsp70/110 Family and G Protein Family

**Figure 10:**
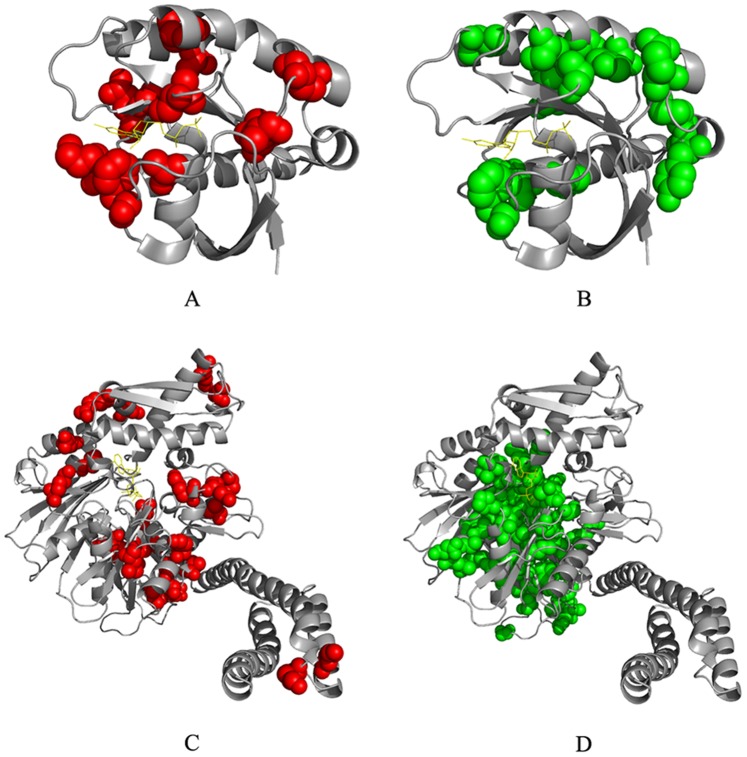
Protein sectors of G protein family (A and B) and Hsp70/110 family (C and D). Red balls and Green balls represent different protein sectors in protein 3D structure. Residues comprising protein sectors are displayed in space filling representation with a van der Waals surface.

To demonstrate the generality of the algorithm BIFANR, we carried out protein sector prediction for another 2 protein families: Hsp70/110 family and G protein family. For the Hsp70/110 family, the MSA consists of 926 sequences and 605 positions, and for the G protein family the MSA consists of 678 sequences and 160 positions. BIFANR detected 2 significant protein sectors for each of the two families (see Figure 10 and Table S2 ). The internal correlation test and the statistical independence test for both datasets showed that the conclusions drawn from the experiments on Hsp70/110 family and G protein family were also well supported (Fig S1 and S2).

## Discussion

Exploring the coevolved protein sectors among homologous proteins is currently a hot issue, especially for the studies of the biological features and evolutionary direction of proteins. There have been a few methods developed for detecting coevolved sites in a protein family but they all suffer from low accuracy and low robustness. In this paper, we proposed a new algorithm BIFANR aiming to address these issues.

BIFANR is unique in the following aspects. First, BIFANR has a noise reduction step for the sites in MSA. This step can reduce the complexity of the calculation and improve the accuracy. Second, motivated by factor analysis, a stochastic simulation step is adopted to choose non-random eigenvectors. This step ensures that the protein sectors detected are non-random and thus of high credibility. Third, BIFANR uses varimax orthogonal rotation to calculate the linear combination of selected eigenvectors, which leads to the significant statistical independence between protein sectors. Fourth, the algorithm avoids manual curation, such as visual inspecting and screening, thus is more practical to use for high throughput analysis.

Besides, BIFANR is robust for various data scales. When the data is randomly reduced to half in size, the result remains almost the same. We did this on both S1A and PDZ family and compared the new results with the old ones. As shown in Table 2 and Table 3, the accuracy remained high especially for PDZ family, which indicates that BIFANR is robust for data scales.

**Table 2:** The performance of BIFANR on original data and half data in S1A family.

	Original data	Half data
**sector1**	95%	90%
**sector2**	92%	88%
**sector3**	90.91%	90.91%

**Table 3:** The performance of BIFANR on original data and half data in PDZ family.

	Original data	Half data
**sector1**	100%	100%
**sector2**	90.91%	90.91%

In the future, we will consider using the common amino acid substitution matrix (e.g. PAM or BLOSUM) to incorporate the relationships among amino acids, as currently BIFANR assumes that all the 20 amino acids are independent. In addition, we will work with biologists to use our predicted sectors to guide site-specific mutagenesis experiments on some selected genes of interests.

## Materials and Methods

### 1. Obtaining Materials

In this study, we chose the classic S1A serine protease family and PDZ family for protein sector analysis (see Data S1.zip) as R. Ranganathan did in previous study [7]. The members of S1A family have the same peptide bond hydrolysis mechanism and possess broad substrate spectrum. PDZ family is a common domain in signal protein, widely existing in bacteria, fungi, plant, animal, and virus [51], [52], [53], [54], which mediates the protein-protein interaction between α_2_–β_2_ groove and the C-terminal ligand of target protein. The dataset was obtained through PSI-BLAST [55] from NCBI (release 2.2.14, May-07-2006) non-redundant database with 3TGI and 1BE9 as the template sequences, and the multiple sequence alignment was provided by Clustal X [7], [56].

### 2. Construction of Weighted Covariance Matrix and Reduction of Noise Sites

#### 2.1 Construction of covariance matrix

Proteins consist of 20 common amino acids. For the purpose of calculation, we replace 20 common amino acids with number 1–20 and gap with 0.

BIFANR constructs the covariance matrix by the formula:
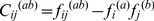
(1)in which 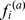 is the observed frequency of the amino acid *a* at position *i* and 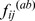 represents the joint frequency of having *a* at position *i* and *b* at position *j*.

BIFANR constructs the weighted covariance matrix 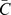 by the formula:.
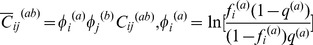
(2)

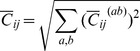
(3)where 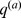 represents the background frequency of amino acid *a*. 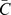 is the weighted covariance matrix. The more relevant between sites *i* and *j*, the higher probability of synergistic reaction and the larger correlation coefficient; On the contrary, the correlation coefficient is small.

#### 2.2 Reduction of noise sites

In this study, we consider that every two sites in one protein sector have significant correlation, thus have large correlation coefficient. For each site *i*, we take top 5% sites that have the largest correlation coefficients with *i*. And we calculate the average of them and represent this average with *Rmax*(*i*),. Then, we sum *Rmax*(*i*) for all sites *i* and calculate its average, represented with *plus*. If site *i* belongs to one protein sector, its *Rmax*(*i*) should be larger than 0.8*plus*. Thus, the site with *Rmax* no larger than 0.8*plus* is considered as noise site. Finally, we remove the rows and columns of noise sites in 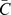 and represent the new matrix with 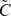.

### 3. Bi-factor Analysis

#### 3.1. Selection of eigenvectors

In this study, we selected eigenvectors by the following steps. (1) Calculate eigenvalues 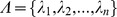 of the matrix 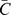 in descending order, where *n* is the number of amino acid sites. (2) Scramble each column of the original alignment randomly and independently, and represent the new random alignment with *Al*. Then we calculate the eigenvalues of *Al.* After randomly combining for 100 times, we obtain 100n eigenvalues and put them into set *E*. (3) Count the number of eigenvalues in *E* which are bigger than 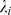 in 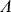 and represent this number with *N*(*i*), Obviously, *N*(*i*) is in increasing order for *i* = 1, …, *n.* (4) Let *N*(*t*) be the last one less than 100 in {*N*(*i*); *i* = 1, …, *n* } and take the corresponding eigenvectors of 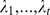.

#### 3.2 Rotation, the linear combination of eigenvectors

We conduct varimax orthogonal rotation which can maximum [57]:
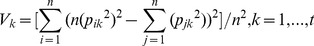
(4)where is 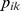 the *i*-th element of the *k*-th eigenvector after rotation.

#### 3.3 Bidirectional selection of protein sectors

We construct protein sectors for each factor (eigenvector after rotation) as following. (1) Put factor coefficients of this factor in descending order, select top 50% and calculate the average of them, represented with *w*. Then we calculate the ratio 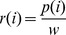, where *p*(*i*) is the coefficient between the factor and amino acid site *i*, i.e. the *i*-th sector of the factor. (2) If the ratio *r*(*i*) is not smaller than the given threshold 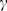 and *p*(*i*) is bigger than the given threshold 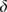, amino acid site *i* belongs to the protein sector. (3) Besides, if *p*(*i*) is large enough, say no smaller than the given threshold 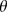, then this amino acid belongs to the protein sector, too.

#### 3.4 Merging of protein sectors

Bidirectional selection of protein sectors may lead to the occurrence of overlap protein sectors and we merge them into one as following. (1) For two overlap protein sectors, we use a vector named *Ssame* to record the overlap sites and another vector *Sdiff* to record the symmetric set difference. (2) Select such site from *Sdiff* satisfying that the sum of the correlation coefficients between the site and sites in *Ssame* is the biggest, and put it into *Ssame*. (3) Repeat step (2) until *Ssame* reaches the size of the smaller protein sector before merging. Then we output *Ssame* as protein sector.

### 4. Examination of Protein Sectors

#### 4.1 Correlations between amino acids in a protein sector

We calculate the average correlation coefficients and random average correlation coefficients of each protein sector. We use these two parameters to measure the significant correlation between amino acids in a protein sector.

#### 4.2 Statistical independence

We use MDI entropy to measure the degree to which a selected group of residues are statistically coupled to each other in the multiple sequence alignment. In this study, the definition of statistical independence is that, if two protein sectors are independent, then the MDI entropy of two taken together must be the sum of their MDI entropies taken individually [7], [58]. We adapt generalized iterative scaling algorithm to calculate MDI entropy [59].

#### 4.3 Calculation of the evolutionary rate

In this study, the evolutionary rate is estimated by Rate4site. Rate4Site adapts maximum likelihood criterion to estimate the normalized rate of evolution at each site, taking into consideration the topology and branch lengths of the phylogenetic tree. The sites with positive values evolve faster than average, and sites with negative values evolve slower than average for that protein.

#### 4.4 Evolutionary independence

To evaluate the evolutionary independence of protein sectors, we use Principle Component Analysis to separate proteins based on the sequence similarities of sites in protein sector.

Programs of algorithm BIFANR can be obtained from Program S1 i.

## Supporting Information

Figure S1 **Average correlation coefficients of protein sectors.** A: Average correlation coefficient of each protein sector in G protein family. B: Average correlation coefficient of each protein sector in Hsp70/110 family. Blue and green columns represent average correlation coefficient of protein sector 1 and protein sector 2 in G protein family. Red and green columns represent average correlation coefficient of protein sector 1 and protein sector 2 in Hsp70/110 family. Black column represents stochastic expected average correlation coefficient.
(TIF)

Figure S2 **Statistical independence of protein sectors.** A: Statistical independence of protein sectors in G protein family. B: Statistical independence of protein sectors in Hsp70/110 family. Red column represents MDI entropy of protein sector 1. Blue column represents MDI entropy of protein sector 2. Black column represents MDI entropy of two protein sectors as a whole. Yellow column represents stochastic expected MDI entropy after disrupting the amino acid sites within two protein sectors 100 times.
(TIF)

Table S1 **The amino acid sites in each protein sector of the two protein families S1A and PDZ.**
(DOC)

Table S2 **The amino acid sites in each protein sector of G protein family and Hsp70/110 family.**
(DOC)

Program S1 **Programs of algorithm BIFANR.**
(ZIP)

Data S1 **Data of S1A serine protease family, PDZ family, HSP family and G family.**
(ZIP)

## References

[1] Chakrabarti S, Panchenko AR (2010) Structural and functional roles of coevolved sites in proteins. PLoS One 5: e8591. 20066038 10.1371/journal.pone.0008591 PMC2797611

[2] Little DY, Chen L (2009) Identification of coevolving residues and coevolution potentials emphasizing structure, bond formation and catalytic coordination in protein evolution. PLoS One 4: e4762. 19274093 10.1371/journal.pone.0004762 PMC2651771

[3] Volff J-N (2007) Gene and protein evolution. Basel; New York: Karger. vii, 194 p. p.

[4] Bowie JU, Reidhaar-Olson JF, Lim WA, Sauer RT (1990) Deciphering the message in protein sequences: tolerance to amino acid substitutions. Science 247: 1306–1310. 2315699 10.1126/science.2315699

[5] Lesk AM, Chothia C (1982) Evolution of proteins formed by beta-sheets. II. The core of the immunoglobulin domains. J Mol Biol 160: 325–342. 7175935 10.1016/0022-2836(82)90179-6

[6] Chothia C, Lesk AM (1982) Evolution of proteins formed by beta-sheets. I. Plastocyanin and azurin. J Mol Biol 160: 309–323. 6816943 10.1016/0022-2836(82)90178-4

[7] Halabi N, Rivoire O, Leibler S, Ranganathan R (2009) Protein sectors: evolutionary units of three-dimensional structure. Cell 138: 774–786. 19703402 10.1016/j.cell.2009.07.038 PMC3210731

[8] Buck MJ, Atchley WR (2005) Networks of coevolving sites in structural and functional domains of serpin proteins. Mol Biol Evol 22: 1627–1634. 15858204 10.1093/molbev/msi157

[9] Du QS, Wang CH, Liao SM, Huang RB (2010) Correlation analysis for protein evolutionary family based on amino acid position mutations and application in PDZ domain. PLoS One 5: e13207. 20949088 10.1371/journal.pone.0013207 PMC2950854

[10] Nimrod G, Glaser F, Steinberg D, Ben-Tal N, Pupko T (2005) In silico identification of functional regions in proteins. Bioinformatics 21 Suppl 1i328–337. 15961475 10.1093/bioinformatics/bti1023

[11] Oliveira L, Paiva PB, Paiva AC, Vriend G (2003) Identification of functionally conserved residues with the use of entropy-variability plots. Proteins 52: 544–552. 12910454 10.1002/prot.10490

[12] Oliveira L, Paiva AC, Vriend G (2002) Correlated mutation analyses on very large sequence families. Chembiochem 3: 1010–1017. 12362367 10.1002/1439-7633(20021004)3:10<1010::AID-CBIC1010>3.0.CO;2-T

[13] Friedberg I, Margalit H (2002) Persistently conserved positions in structurally similar, sequence dissimilar proteins: roles in preserving protein fold and function. Protein Sci 11: 350–360. 11790845 10.1110/ps.18602 PMC2373454

[14] Travers SA, Fares MA (2007) Functional coevolutionary networks of the Hsp70-Hop-Hsp90 system revealed through computational analyses. Mol Biol Evol 24: 1032–1044. 17267421 10.1093/molbev/msm022

[15] Fares MA, Travers SA (2006) A novel method for detecting intramolecular coevolution: adding a further dimension to selective constraints analyses. Genetics 173: 9–23. 16547113 10.1534/genetics.105.053249 PMC1461439

[16] Kim Y, Koyuturk M, Topkara U, Grama A, Subramaniam S (2006) Inferring functional information from domain co-evolution. Bioinformatics 22: 40–49. 16301205 10.1093/bioinformatics/bti723

[17] Codoner FM, Fares MA, Elena SF (2006) Adaptive covariation between the coat and movement proteins of prunus necrotic ringspot virus. J Virol 80: 5833–5840. 16731922 10.1128/JVI.00122-06 PMC1472603

[18] Martin LC, Gloor GB, Dunn SD, Wahl LM (2005) Using information theory to search for co-evolving residues in proteins. Bioinformatics 21: 4116–4124. 16159918 10.1093/bioinformatics/bti671

[19] Pazos F, Helmer-Citterich M, Ausiello G, Valencia A (1997) Correlated mutations contain information about protein-protein interaction. J Mol Biol 271: 511–523. 9281423 10.1006/jmbi.1997.1198

[20] Chelvanayagam G, Eggenschwiler A, Knecht L, Gonnet GH, Benner SA (1997) An analysis of simultaneous variation in protein structures. Protein Eng 10: 307–316. 9194155 10.1093/protein/10.4.307

[21] Atwell S, Ultsch M, De Vos AM, Wells JA (1997) Structural plasticity in a remodeled protein-protein interface. Science 278: 1125–1128. 9353194 10.1126/science.278.5340.1125

[22] Taylor WR, Hatrick K (1994) Compensating changes in protein multiple sequence alignments. Protein Eng 7: 341–348. 8177883 10.1093/protein/7.3.341

[23] Codoner FM, Fares MA (2008) Why should we care about molecular coevolution? Evol Bioinform Online 4: 29–38. 19204805 PMC2614197

[24] Pei J, Cai W, Kinch LN, Grishin NV (2006) Prediction of functional specificity determinants from protein sequences using log-likelihood ratios. Bioinformatics 22: 164–171. 16278237 10.1093/bioinformatics/bti766

[25] Choi SS, Li W, Lahn BT (2005) Robust signals of coevolution of interacting residues in mammalian proteomes identified by phylogeny-aided structural analysis. Nat Genet 37: 1367–1371. 16282975 10.1038/ng1685

[26] Pollock DD, Taylor WR, Goldman N (1999) Coevolving protein residues: maximum likelihood identification and relationship to structure. J Mol Biol 287: 187–198. 10074416 10.1006/jmbi.1998.2601

[27] Dimmic MW, Hubisz MJ, Bustamante CD, Nielsen R (2005) Detecting coevolving amino acid sites using Bayesian mutational mapping. Bioinformatics 21 Suppl 1i126–135. 15961449 10.1093/bioinformatics/bti1032

[28] Fukami-Kobayashi K, Schreiber DR, Benner SA (2002) Detecting compensatory covariation signals in protein evolution using reconstructed ancestral sequences. J Mol Biol 319: 729–743. 12054866 10.1016/S0022-2836(02)00239-5

[29] Fares MA, McNally D (2006) CAPS: coevolution analysis using protein sequences. Bioinformatics 22: 2821–2822. 17005535 10.1093/bioinformatics/btl493

[30] Johnson RA, Wichern DW (1988) Applied multivariate statistical analysis. Englewood Cliffs, N.J.: Prentice-Hall. xvi, 607 p. p.

[31] Lockless SW, Ranganathan R (1999) Evolutionarily conserved pathways of energetic connectivity in protein families. Science 286: 295–299. 10514373 10.1126/science.286.5438.295

[32] Pollock DD, Taylor WR (1997) Effectiveness of correlation analysis in identifying protein residues undergoing correlated evolution. Protein Eng 10: 647–657. 9278277 10.1093/protein/10.6.647

[33] Pasternak A, Ringe D, Hedstrom L (1999) Comparison of anionic and cationic trypsinogens: the anionic activation domain is more flexible in solution and differs in its mode of BPTI binding in the crystal structure. Protein Sci 8: 253–258. 10210204 10.1110/ps.8.1.253 PMC2144100

[34] Pupko T, Bell RE, Mayrose I, Glaser F, Ben-Tal N (2002) Rate4Site: an algorithmic tool for the identification of functional regions in proteins by surface mapping of evolutionary determinants within their homologues. Bioinformatics 18 Suppl 1S71–77. 12169533 10.1093/bioinformatics/18.suppl_1.s71

[35] Doyle DA, Lee A, Lewis J, Kim E, Sheng M, et al (1996) Crystal structures of a complexed and peptide-free membrane protein-binding domain: molecular basis of peptide recognition by PDZ. Cell 85: 1067–1076. 8674113 10.1016/s0092-8674(00)81307-0

[36] Hedstrom L (2002) Serine protease mechanism and specificity. Chem Rev 102: 4501–4524. 12475199 10.1021/cr000033x

[37] Wang EC, Hung SH, Cahoon M, Hedstrom L (1997) The role of the Cys191-Cys220 disulfide bond in trypsin: new targets for engineering substrate specificity. Protein Eng 10: 405–411. 9194165 10.1093/protein/10.4.405

[38] Hedstrom L (1996) Trypsin: a case study in the structural determinants of enzyme specificity. Biol Chem 377: 465–470. 8922280

[39] Perona JJ, Craik CS, Fletterick RJ (1993) Locating the catalytic water molecule in serine proteases. Science 261: 620–622. 8342029 10.1126/science.8342029

[40] Craik CS, Largman C, Fletcher T, Roczniak S, Barr PJ, et al (1985) Redesigning trypsin: alteration of substrate specificity. Science 228: 291–297. 3838593 10.1126/science.3838593

[41] Lee WS, Park CH, Byun SM (2004) Streptomyces griseus trypsin is stabilized against autolysis by the cooperation of a salt bridge and cation-pi interaction. J Biochem 135: 93–99. 14999014 10.1093/jb/mvh011

[42] Bodi A, Kaslik G, Venekei I, Graf L (2001) Structural determinants of the half-life and cleavage site preference in the autolytic inactivation of chymotrypsin. Eur J Biochem 268: 6238–6246. 11733020 10.1046/j.0014-2956.2001.02578.x

[43] Baird TT Jr, Wright WD, Craik CS (2006) Conversion of trypsin to a functional threonine protease. Protein Sci 15: 1229–1238. 16672242 10.1110/ps.062179006 PMC2242550

[44] Huntington JA, Esmon CT (2003) The molecular basis of thrombin allostery revealed by a 1.8 A structure of the “slow” form. Structure 11: 469–479. 12679024 10.1016/s0969-2126(03)00049-2

[45] Guinto ER, Caccia S, Rose T, Futterer K, Waksman G, et al (1999) Unexpected crucial role of residue 225 in serine proteases. Proc Natl Acad Sci U S A 96: 1852–1857. 10051558 10.1073/pnas.96.5.1852 PMC26700

[46] Coleman SK, Cai C, Kalkkinen N, Korpi ER, Keinanen K (2010) Analysis of the potential role of GluA4 carboxyl-terminus in PDZ interactions. PLoS One 5: e8715. 20090852 10.1371/journal.pone.0008715 PMC2806832

[47] Peterson FC, Penkert RR, Volkman BF, Prehoda KE (2004) Cdc42 regulates the Par-6 PDZ domain through an allosteric CRIB-PDZ transition. Mol Cell 13: 665–676. 15023337 10.1016/s1097-2765(04)00086-3

[48] Ostergaard H, Tachibana C, Winther JR (2004) Monitoring disulfide bond formation in the eukaryotic cytosol. J Cell Biol 166: 337–345. 15277542 10.1083/jcb.200402120 PMC2172265

[49] Hanson GT, Aggeler R, Oglesbee D, Cannon M, Capaldi RA, et al (2004) Investigating mitochondrial redox potential with redox-sensitive green fluorescent protein indicators. J Biol Chem 279: 13044–13053. 14722062 10.1074/jbc.M312846200

[50] Mishra P, Socolich M, Wall MA, Graves J, Wang Z, et al (2007) Dynamic scaffolding in a G protein-coupled signaling system. Cell 131: 80–92. 17923089 10.1016/j.cell.2007.07.037

[51] Boxus M, Twizere JC, Legros S, Dewulf JF, Kettmann R, et al (2008) The HTLV-1 Tax interactome. Retrovirology 5: 76. 18702816 10.1186/1742-4690-5-76 PMC2533353

[52] Carmena A, Speicher S, Baylies M (2006) The PDZ protein Canoe/AF-6 links Ras-MAPK, Notch and Wingless/Wnt signaling pathways by directly interacting with Ras, Notch and Dishevelled. PLoS One 1: e66. 17183697 10.1371/journal.pone.0000066 PMC1762375

[53] Walsh NP, Alba BM, Bose B, Gross CA, Sauer RT (2003) OMP peptide signals initiate the envelope-stress response by activating DegS protease via relief of inhibition mediated by its PDZ domain. Cell 113: 61–71. 12679035 10.1016/s0092-8674(03)00203-4

[54] Ponting CP (1997) Evidence for PDZ domains in bacteria, yeast, and plants. Protein Sci 6: 464–468. 9041651 10.1002/pro.5560060225 PMC2143646

[55] Altschul SF, Madden TL, Schaffer AA, Zhang J, Zhang Z, et al (1997) Gapped BLAST and PSI-BLAST: a new generation of protein database search programs. Nucleic Acids Res 25: 3389–3402. 9254694 10.1093/nar/25.17.3389 PMC146917

[56] Thompson JD, Gibson TJ, Plewniak F, Jeanmougin F, Higgins DG (1997) The CLUSTAL_X windows interface: flexible strategies for multiple sequence alignment aided by quality analysis tools. Nucleic Acids Res 25: 4876–4882. 9396791 10.1093/nar/25.24.4876 PMC147148

[57] Xie X (1989) Yin su fen xi : yi zhong ke xue yan jiu di gong ju. Peking: Zhongguo she hui ke xue chu ban she : Xin hua shu dian jing xiao. 2, 4, 192 p., 191 folded leaf of plates p.

[58] Kullback S (1997) Information theory and statistics. Mineola, N.Y.: Dover Publications. xv, 399 p. p.

[59] Darroch JN, Ratcliff D (1972) Generalized iterative scaling for log-linear models. The annals of mathematical statistics 43: 1470–1480.

