# Supplementary figures and images for: Bi-Factor Analysis Based on Noise-Reduction (BIFANR): A New Algorithm for Detecting Coevolving Amino Acid Sites in Proteins
Source: PLoS One. 2013 Nov 20;8(11):e79764. doi: 10.1371/journal.pone.0079764 (PMC3835919; doi:10.1371/journal.pone.0079764)

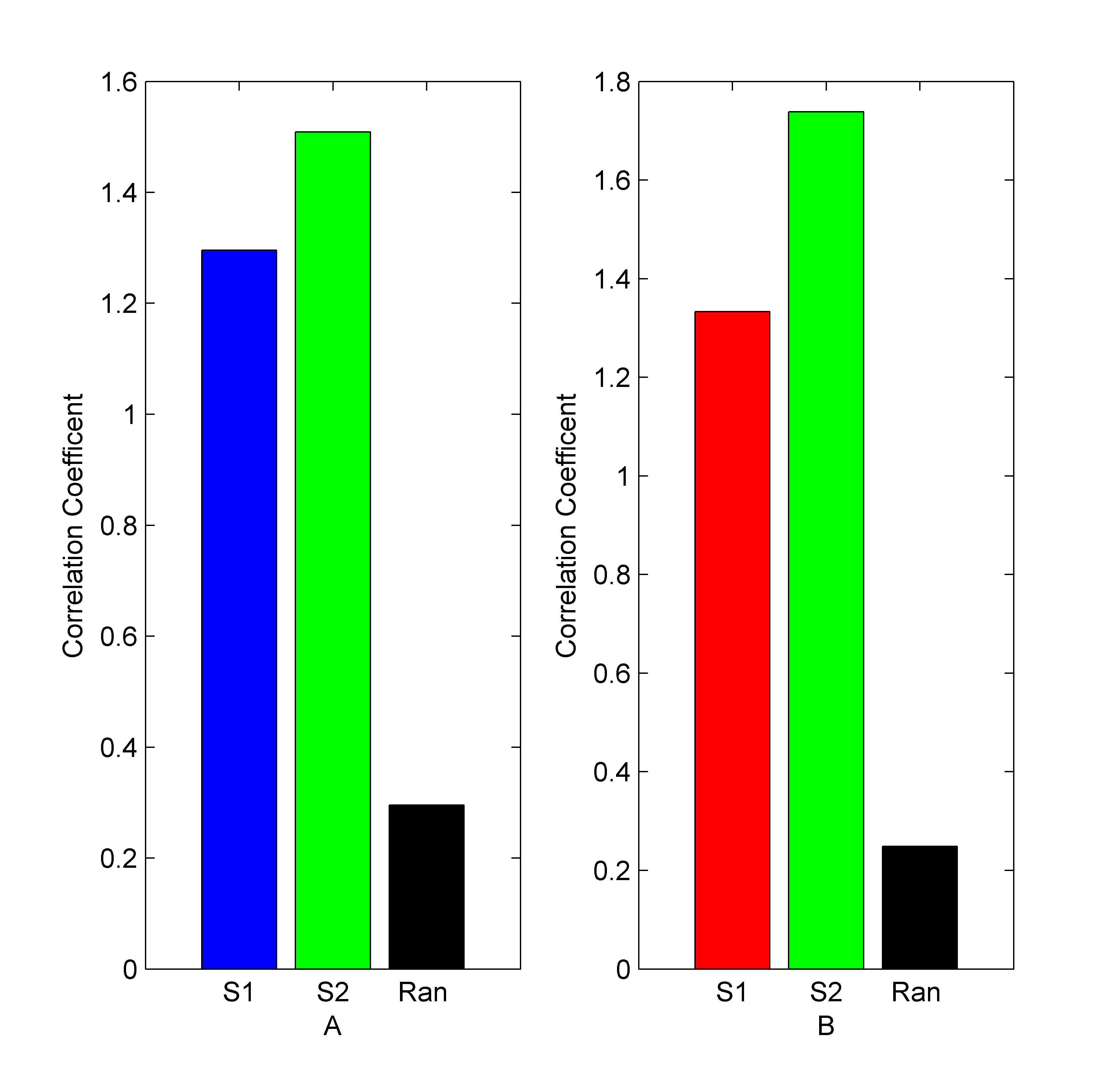

Supplement: Figure S1 — Average correlation coefficients of protein sectors. A: Average correlation coefficient of each protein sector in G protein family. B: Average correlation coefficient of each protein sector in Hsp70/110 family. Blue and green columns represent average correlation coefficient of protein sector 1 and protein sector 2 in G protein family. Red and green columns represent average correlation coefficient of protein sector 1 and protein sector 2 in Hsp70/110 family. Black column represents stochastic expected average correlation coefficient. (TIF) [file pone.0079764.s001.tif]

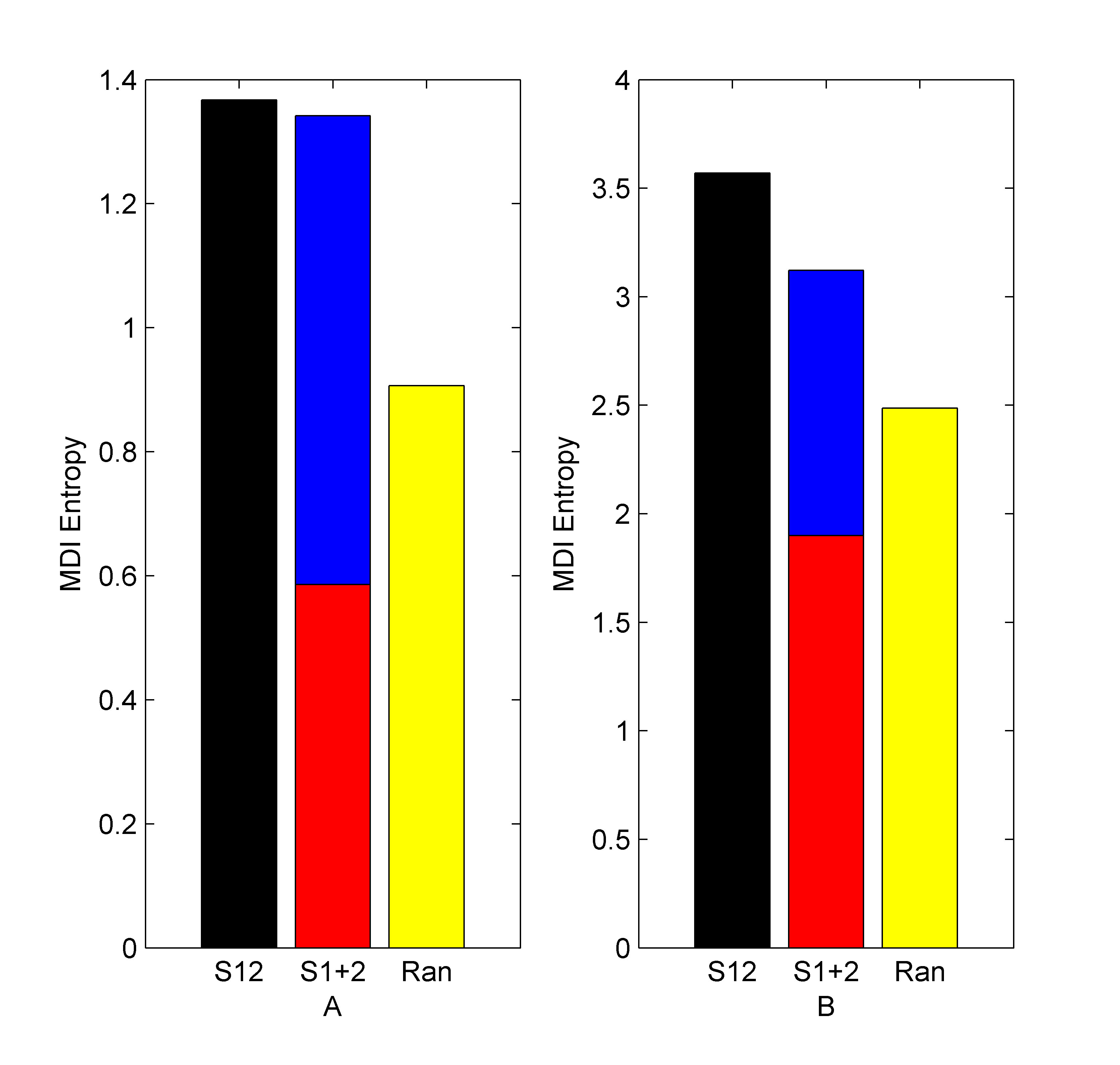

Supplement: Figure S2 — Statistical independence of protein sectors. A: Statistical independence of protein sectors in G protein family. B: Statistical independence of protein sectors in Hsp70/110 family. Red column represents MDI entropy of protein sector 1. Blue column represents MDI entropy of protein sector 2. Black column represents MDI entropy of two protein sectors as a whole. Yellow column represents stochastic expected MDI entropy after disrupting the amino acid sites within two protein sectors 100 times. (TIF) [file pone.0079764.s002.tif]
