# Supplementary material for: Bi-Factor Analysis Based on Noise-Reduction (BIFANR): A New Algorithm for Detecting Coevolving Amino Acid Sites in Proteins
Source: PLoS One. 2013 Nov 20;8(11):e79764. doi: 10.1371/journal.pone.0079764 (PMC3835919; doi:10.1371/journal.pone.0079764)
Supplement: Table S1 — The amino acid sites in each protein sector of the two protein families S1A and PDZ. (DOC) [file pone.0079764.s003.doc]

**Table S1.** The amino acid sites in each protein sector of the two protein families S1A and PDZ.

| **S1A (3TGI)** | | | **PDZ (1BE9)** | |
| --- | --- | --- | --- | --- |
| **Sector 1** | **Sector 2** | **Sector 3** | **Sector 1** | **Sector 2** |
| 17 | 21 | 19 | 322 | 323 |
| 30 | 26 | 33 | 325 | 327 |
| 111 | 46 | 42 | 330 | 336 |
| 161 | 52 | 43 | 347 | 341 |
| 172 | 68 | 55 | 353 | 345 |
| 176 | 69 | 56 | 372 | 350 |
| 177 | 71 | 57 |  | 351 |
| 180 | 77 | 58 |  | 352 |
| 183 | 81 | 102 |  | 359 |
| 184 | 104 | 142 |  | 364 |
| 187 | 105 | 182 |  | 375 |
| 188 | 107 | 194 |  | 379 |
| 189 | 108 | 195 |  |  |
| 191 | 123 | 196 |  |  |
| 192 | 124 | 197 |  |  |
| 213 | 133 | 198 |  |  |
| 215 | 136 | 199 |  |  |
| 216 | 153 | 213 |  |  |
| 221 | 157 | 214 |  |  |
| 226 | 201 | 216 |  |  |
| 227 | 203 | 225 |  |  |
| 228 | 210 | 231 |  |  |
| 230 | 237 |  |  |  |
|  | 242 |  |  |  |
|  | 245 |  |  |  |
