# Supplementary material for: Bi-Factor Analysis Based on Noise-Reduction (BIFANR): A New Algorithm for Detecting Coevolving Amino Acid Sites in Proteins
Source: PLoS One. 2013 Nov 20;8(11):e79764. doi: 10.1371/journal.pone.0079764 (PMC3835919; doi:10.1371/journal.pone.0079764)
Supplement: Table S2 — The amino acid sites in each protein sector of G protein family and Hsp70/110 family. (DOC) [file pone.0079764.s004.doc]

**Table S2.** The amino acid sites in each protein sector of G protein family and Hsp70/110 family.

| **G (1Q21)** | | **Hsp70/110 (DnaK-Sse1Model)** | | |
| --- | --- | --- | --- | --- |
| **Sector1** | **Sector2** | **Sector1** | **Sector2** | |
| 14 | 5 | 27 | 7 | 375 |
| 18 | 11 | 41 | 9 | 378 |
| 28 | 28 | 55 | 11 | 391 |
| 58 | 61 | 57 | 12 | 394 |
| 59 | 71 | 61 | 14 | 396 |
| 62 | 72 | 122 | 38 | 397 |
| 90 | 89 | 124 | 112 | 399 |
| 115 | 93 | 135 | 116 | 400 |
| 134 | 96 | 147 | 139 | 402 |
| 143 | 113 | 160 | 141 | 405 |
| 147 | 125 | 168 | 144 | 406 |
|  | 151 | 169 | 145 | 415 |
|  | 152 | 245 | 146 | 426 |
|  | 156 | 251 | 154 | 428 |
|  |  | 302 | 165 | 431 |
|  |  | 311 | 171 | 433 |
|  |  | 319 | 172 | 440 |
|  |  | 350 | 175 | 443 |
|  |  | 363 | 178 | 444 |
|  |  | 411 | 181 | 445 |
|  |  | 432 | 182 | 450 |
|  |  | 455 | 195 | 454 |
|  |  | 483 | 197 | 457 |
|  |  | 501 | 198 | 459 |
|  |  | 556 | 199 | 462 |
|  |  | 562 | 200 | 463 |
|  |  |  | 201 | 468 |
|  |  |  | 218 | 474 |
|  |  |  | 223 | 479 |
|  |  |  | 227 | 482 |
|  |  |  | 231 | 486 |
|  |  |  | 344 | 488 |
|  |  |  | 367 | 494 |
|  |  |  | 371 |  |
